# Progressive colonic stenosis in an infant: Successful treatment with endoscopic balloon dilation

**DOI:** 10.1002/jpr3.12114

**Published:** 2024-07-28

**Authors:** Korppong Plubjang, Kanticha Chatpermporn, Nimmita Srisan, Paisarn Vejchapipat, Teerasak Phewplung, Atchara Mahayosnond, Palittiya Sintusek

**Affiliations:** ^1^ Department of Pediatrics, King Chulalongkorn Memorial Hospital, The Thai Red Cross Society, Faculty of Medicine Chulalongkorn University Bangkok Thailand; ^2^ Department of Pediatrics, Division of Gastroenterology, Center of Excellence in Thai Pediatric Gastroenterology, Hepatology and Immunology (TPGHAI), King Chulalongkorn Memorial Hospital, The Thai Red Cross Society, Faculty of Medicine Chulalongkorn University Bangkok Thailand; ^3^ Department of Surgery, King Chulalongkorn Memorial Hospital, The Thai Red Cross Society, Faculty of Medicine Chulalongkorn University Bangkok Thailand; ^4^ Department of Radiology, King Chulalongkorn Memorial Hospital, The Thai Red Cross Society, Faculty of Medicine Chulalongkorn University Bangkok Thailand

**Keywords:** colonic atresia, constipation, endoscopic dilation, lower gut obstruction, novel

## Abstract

Acquired colonic stenosis is extremely rare in infants and surgical resection is the mainstay of treatment. Endoscopic balloon dilation has shown success in treating bowel stenosis from inflammatory bowel disease but its application in infants with colonic strictures of other origin has not been widely explored. We report a 4‐week‐old male infant who developed significant abdominal distension due to progressive colonic stenosis, occurring 2 weeks following balloon valvuloplasty for his severe valvular pulmonary stenosis. The progressive colonic stenosis was successfully managed through endoscopic balloon dilation. Following this procedure, he exhibited clinical improvement, with subsequent imaging revealing no remaining stricture. Over the 16‐month follow‐up period, no clinical features suggestive of constipation or lower gut obstruction were observed. This case serves as evidence that endoscopic balloon dilation is a promising and safe therapeutic option for treating colonic stenosis in infants.

## 1 INTRODUCTION

Colonic stenosis is an extremely rare condition that can be categorized into two types: congenital and acquired.^1^ The etiology of colonic stenosis can indeed involve compromised blood supply to the colon, stemming from various factors during pregnancy, such as emboli, vasoconstriction due to medications, vessel compression, or postnatal events like necrotizing enterocolitis.^2^ Recently advanced endoscopy has replaced the standard surgical resection of colonic stenosis complicated by inflammatory bowel disease in adult and a few children resulting in favorable outcomes.^3^, ^4^, ^5^ While recently advanced endoscopy is now the standard management for colonic strictures in inflammatory bowel disease,^6^ surgery remains the management of choice for other conditions, particularly, the younger patients. We have reported, for the first time, the successful use of endoscopic balloon dilation in treating progressive colonic stenosis in an infant who had previously undergone a percutaneous balloon pulmonary valvuloplasty procedure for severe valvular pulmonary stenosis.

## 2 CLINICAL CASE PRESENTATION

**Figure 1:**
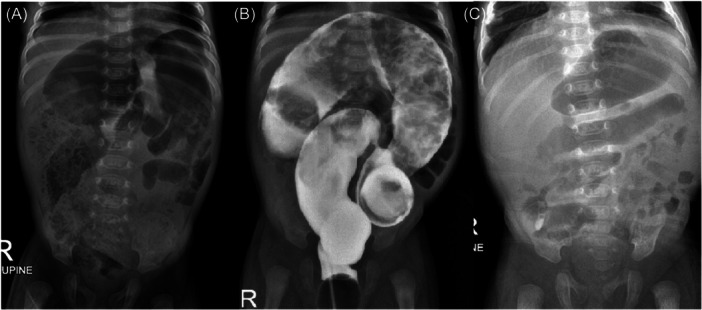
A portable anteroposterior supine abdominal radiograph of the 6‐week‐old infant (A) revealed diffuse colonic dilatation with moderate amount of fecal material in colon, more pronounce at right‐side colon (allows); (B) after bedside barium enema, showed a focal severe luminal narrowing (arrow) of the proximal sigmoid colon proximal colonic dilatation and retained fecal content. (C) after conservative treatment, revealed interval decreasing degree of the colonic dilatation as well as a much‐decreasing amount of the retained fecal material. Noted no abnormal small bowel dilatation. R, right side.

We present a case of colonic stenosis in a male infant born preterm at 34 weeks, with a birth weight of 2055 g, who underwent cesarean section due to nonreassuring fetal conditions. The infant was transferred from a secondary hospital to our tertiary hospital due to presenting with central cyanosis and respiratory distress since birth, attributed to a severe valvular pulmonary stenosis and patent ductus arteriosus. At 15 days of age, a percutaneous balloon pulmonary valvuloplasty was successfully performed, and the infant was discharged from the hospital at 3 weeks of age. However, at 4 weeks of age, he presented with progressive abdominal distension and fever, resulting in readmission at 6 weeks of age due to enterocolitis (Figure 1A). A bedside barium enema performed by a pediatric surgeon identified a suspected stenosis point at the sigmoid colon (Figure 1B). However, following conservative treatment with antibiotics, rectal enemas, and nil per mouth for 7 days, the abdominal distension subsided, allowing for a gradual increase in feeding until full feeding was achieved in 4 days. An abdominal X‐ray demonstrated the subsequently improvement of the dilated colon (Figure 1C). Consequently, the patient was discharged from hospital with a plan for regular follow‐up.

**Figure 2:**
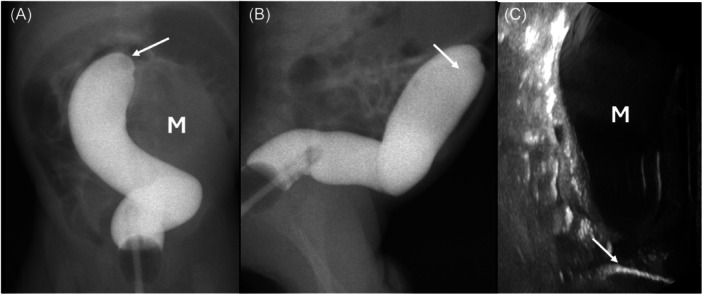
A high‐pressure contrast enema on anteroposterior (A) and lateral view (B) exhibited a blind‐ended sigmoid colon (arrow) with a mass‐like soft tissue density at left‐sided sigmoid colon (M in A). Additional ultrasound (C) revealed a fluid‐filled bowel loop proximal to the blinded end (arrow) of the sigmoid colon (M in C), suggestive of sigmoid colonic obstruction.

One week after being discharged, the patient experienced progressive abdominal distension and reported no bowel movements for 2 days. As a result, he was readmitted for further investigation of the cause of obstruction. Barium enema and abdominal ultrasound revealed a stenosis at the sigmoid colon, where the contrast was unable to pass through the narrowed area (Figure 2).

**Figure 3:**
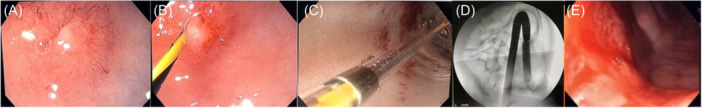
Colonoscopy with balloon dilation. (A) The marked stenotic part of sigmoid colon; (B) guide‐wire was inserted without difficulty; (C) balloon dilation with water‐soluble contrast was performed; (D) the stenotic part disappeared after balloon dilation; (E) the endoscope could pass the stenotic segment, with measured approximately 0.5 cm in length, without difficulty.

After a multidisciplinary discussion, the decision to perform colonoscopy with balloon dilation was initiated (Figure 3). The gastroscope (Olympus GIF‐Q180, diameter 8.8 mm) with CO_2_ insufflation gas was inserted through the rectum, identifying the site of stenosis at the sigmoid, approximately 15 cm from the anus (Figure 3A). A 0.25 mm soft‐tip guidewire was inserted through the stenosis site (Figure 3B), followed by placement of a through‐the‐scope controlled radial release balloon dilator (CRE™ PRO Wireguided 10–12 mm; Boston Scientific) over the guidewire path under fluoroscopy (Figure 3C,D). Afterward, a balloon dilation was performed twice, applying pressures of 4 atm for 1 min and 6 atm for 30 s each time. The stenosis site exhibited dilatation with minimal erythematous mucosa (Figure 3E). Following the procedure, the patient's clinical condition improved, and he was able to pass soft stool every day, experienced relief from abdominal distension, which resumed in regular eating habits within 2 days. A barium enema conducted during the 3‐month follow‐up revealed the absence of any residual colonic stenosis. Subsequent follow‐ups over 13‐month period indicated no recurrence of clinical symptoms suggestive of lower gut obstruction.

## 3 DISCUSSION

We present a case of an infant with progressive abdominal distension after percutaneous balloon pulmonary valvuloplasty from severe pulmonary valve stenosis for which progressive colonic stenosis was the final diagnosis. The information about acquired colonic stenosis exist in the previous literature, especially related to infection‐induced necrotizing enterocolitis^1^ and/or vascular compromise.^2^ There is no definitive evidence for the exact cause in this patient. It is however likely that a mesenteric vascular injury or emboli during a cardiac catheterization procedure might be the casualty.

In infants with clinical manifestations of distal intestinal obstruction, barium enema is frequently the first diagnostic step, significantly aiding in diagnosing Hirschsprung disease,^7^ a condition often associated with distal intestinal obstruction, and can also help in diagnosing colonic stenosis. Additionally, colonoscopy may be used for the diagnosis of colonic stenosis. In our patient, following the first and second barium enema procedures, a suspicious and progressively stenotic point was identified in the distal sigmoid region, prompting the decision to utilize colonoscopy with balloon dilation for treatment. The use of balloon dilatation in pediatric patients with colonic stenosis is relatively uncommon, as surgical intervention is typically the preferred approach.^1^ However, in this particular case, balloon dilation was successfully performed, leading to significant improvement without complications, and the patient was discharged early after treatment. To the best of our knowledge, this is among the first reported cases of balloon dilation successfully used in an infant with progressive colonic stenosis.

Balloon dilation is a commonly performed procedure for conditions affecting the gastrointestinal and urological system.^8^ The risk of perforation is low with balloon dilation because the balloon enables in situ radial dilation without repeated shear forces, which are associated with the risk of false passage and hemorrhage. Additionally, the successful balloon dilation without any complications in this infant might be attributed to the short stenotic segment of the colon and the etiology of the acquired form, which leads to less fibrosis of the stenotic part. Therefore, endoscopic balloon dilation of colonic stenosis in selected cases, even in infants, may represent a novel and minimally invasive procedure that should be considered as one of the therapeutic options.

## 4 CONCLUSION

This case highlights an infant presenting with progressive abdominal distension postpercutaneous balloon pulmonary valvuloplasty, ultimately diagnosed with colonic stenosis. While the exact etiology remains uncertain, suspected causes include mesenteric vascular injury or emboli during cardiac catheterization. Utilizing barium enema and colonoscopy, we identified and successfully treated the stenotic segment via balloon dilation. This report underscores the potential of endoscopic balloon dilation as a minimally invasive therapeutic option for infants with colonic stenosis, particularly in cases with short stenotic segments and acquired etiologies. Further research is warranted to validate its efficacy and safety in similar cases.

## CONFLICT OF INTEREST STATEMENT

The authors declare no conflict of interest.

## References

[1] Xie X , Xiang B , Wu Y , Zhao Y , Wang Q , Jiang X . Infant progressive colonic stenosis caused by antibiotic‐related *Clostridium difficile* colitis—a case report and literature review. BMC Pediatr. 2018;18(1):320. 10.1186/s12887-018-1302-9 30301467 PMC6178272

[2] Erskine JM . Colonic stenosis in the newborn: the possible thromboembolic etiology of intestinal stenosis and atresia. J Pediatr Surg. 1970;5(3):321‐333. 10.1016/0022-3468(70)90189-2 5463651

[3] Foster EN , Quiros JA , Prindiville TP . Long‐term follow‐up of the endoscopic treatment of strictures in pediatric and adult patients with inflammatory bowel disease. J Clin Gastroenterol. 2008;42(8):880‐885. 10.1097/MCG.0b013e3181354440 18645528

[4] Taida T , Nakagawa T , Ohta Y , et al. Long‐term outcome of endoscopic balloon dilatation for strictures in patients with Crohn's disease. Digestion. 2018;98(1):26‐32. 10.1159/000486591 29672285

[5] Bettenworth D , Gustavsson A , Atreja A , et al. A pooled analysis of efficacy, safety, and long‐term outcome of endoscopic balloon dilation therapy for patients with stricturing Crohn's disease. Inflamm Bowel Dis. 2017;23(1):133‐142. 10.1097/MIB.0000000000000988 28002130

[6] Ledder O , Homan M , Furlano R , et al. Approach to endoscopic balloon dilatation in pediatric stricturing crohn disease: a position paper of the endoscopy special interest group of ESPGHAN. J Pediatr Gastroenterol Nutr. 2023;76(6):799‐806. 10.1097/MPG.0000000000003752 36867853

[7] Lourenção PLTA , Valerini FG , Cataneo AJM , et al. Barium enema revisited in the workup for the diagnosis of Hirschsprung's disease. J Pediatr Gastroenterol Nutr. 2019;68(4):e62‐e66. 10.1097/MPG.0000000000002242 30628984

[8] Hidas G , Gibbs D , Alireza A , Khoury AE . Management of rectal stenosis with endoscopic balloon dilatation. J Pediatr Surg. 2013;48(4):e13‐e16. 10.1016/j.jpedsurg.2013.01.035 23583158

